# On‐Chip Chemical Synthesis Using One‐Step 3D Printed Polyperfluoropolyether

**DOI:** 10.1002/cite.202200013

**Published:** 2022-04-25

**Authors:** Andreas Goralczyk, Fadoua Mayoussi, Mario Sanjaya, Santiago Franco Corredor, Sagar Bhagwat, Qingchuan Song, Sarah Schwenteck, Andreas Warmbold, Pegah Pezeshkpour, Bastian E. Rapp

**Affiliations:** ^1^ University of Freiburg Laboratory of Process Technology, NeptunLab Department of Microsystems Engineering (IMTEK) Georges-Köhler-Allee 103 79110 Freiburg im Breisgau Germany; ^2^ University of Freiburg Freiburg Materials Research Center (FMF) Stefan-Meier-Straße 21 79104 Freiburg im Breisgau Germany; ^3^ University of Freiburg FIT Freiburg Center of Interactive Materials and Bioinspired Technologies Georges-Köhler-Allee 105 79110 Freiburg im Breisgau Germany

**Keywords:** Additive manufacturing, Chemical synthesis, Fluorinated materials, Microfluidics

## Abstract

Three‐dimensional (3D) printing has already shown its high relevance for the fabrication of microfluidic devices in terms of precision manufacturing cycles and a wider range of materials. 3D‐printable transparent fluoropolymers are highly sought after due to their high chemical and thermal resistance. Here, we present a simple one‐step fabrication process via stereolithography of perfluoropolyether dimethacrylate. We demonstrate successfully printed microfluidic mixers with 800 µm circular channels for chemistry‐on‐chip applications. The printed chips show chemical, mechanical, and thermal resistance up to 200 °C, as well as high optical transparency. Aqueous and organic reactions are presented to demonstrate the wide potential of perfluoropolyether dimethacrylate for chemical synthesis.

## Graphical abstract

We 3D‐printed microfluidic chips with 800 µm circular channels using stereolithography of perfluoropolyether dimethacrylate. Aqueous and organic reactions are monitored as the reaction of methylene blue with ascorbic acid by visual discoloration of the solution, and the synthesis of N‐benzylidenbenzylamin at ambient conditions by FTIR measurements.

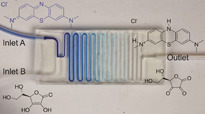

## 1 Introduction

Over the past decades chemical synthesis in microsystems has seen significant interest for a wide range of applications in pharmaceuticals and chemistry taking advantage of continuous flow, controlled mixing, heat and mass transfer, as well as ease of integration 1, 2, 3, 4, 5, 6. The history of chemical synthesis on miniaturized systems goes more than two decades addressing a wide range of applications in security, temperature control, efficient mixing in large vessels and high cost of cooling systems. However, the time and cost of manufacturing scaled‐up microsystems have to be improved 7. Microfluidic devices, developed for numerous applications in biology 8, separation sciences 9, 10, analytical chemistry 3, and medicine 11, are promising platforms for chemical synthesis. These devices are typically capable of handling small volumes of liquids and rapidly carrying out chemical reactions while enabling process automation, low fabrication costs as well as the potential for precision control of concentrations in space and time 12, 13, 14, 15. Jensen et al. reviewed the application of microsystems for chemical synthesis using flow chemistry in academia and industry, aiming at replacing the traditional batch flask processing with tube systems to develop new reactions in sub‐millimeter systems 1. Two types of microsystems include the integrated and component‐based systems, which use either tubes or microstructured devices (microreactors). Tubes are most commonly made of either stainless steel or perfluorinated polymers. Alternatively, microreactors can be machined from glass, silicon‐glass, ceramic, or stainless steel by microfabrication techniques.

Tube‐based system suffer from dispersion in diffusion or poor chemical compatibility due to low heat transfer. The main challenge of microsystems for chemical synthesis is the microfabrication of chemically compatible microchip for integration with valves and pumps. The fabrication of microchips for chemical synthesis is nevertheless still complex, time consuming, and requires access to modern cleanroom facilities. Most of the fabrication approaches involve technologies such as lithography 16, hot embossing 17 or injection moulding 18. Early microfluidic devices were fabricated by micromachining of silicon or glass, which were mostly limited to simple 2D structures as well as requiring the use of hazardous chemicals for wet etching such as, e.g., hydrofluoric acid 19, 20. Owing to its ease of fabrication, low cost and high transparency, polydimethylsiloxane (PDMS) quickly became the standard material for microfluidics, effectively displacing silicon and glass 21, 22. However, PDMS is not a suitable material for chemical reactions (as it heavily swells in most organic solvents) nor for three‐dimensional structuring because it is not directly accessible via 3D printing or advanced manufacturing. Although not a pressing problem in the miniaturization of biological or biochemical assay (which usually require water as solvent), the prevalence of PDMS has been a limiting factor in the miniaturization of chemical synthesis due to its inferior solvent resistance.

As of today, there is a strong lack in materials that are chemically resistant and accessible via 3D printing. To pave the way towards using microfluidics in the miniaturization of chemical synthesis, two essential challenges must be overcome. Firstly, novel materials capable of and suitable for handling organic solvents must be found. Secondly, these materials must be accessible to advanced manufacturing techniques such as high‐resolution 3D printing. 3D printing already had huge impacts on the field of microfluidics enabling fast and high‐resolution fabrication of complex chip designs 18, 19. Using 3D printing, highly‐complex prototypes and small‐series microfluidic chips can be fabricated in a single step based on digital computer‐assisted design (CAD) information and in short amounts of time. Various 3D printing methods including fused deposition modeling (FDM) 23, 24, 25, 26, inkjet printing 27, 28 and stereolithography (SLA) 29, 30 have been reported for the fabrication of microfluidic devices. Several groups reviewed these methods in terms of resolution, costs, and time 13, 31, 32, 33. Among these techniques, SLA remains the method of choice due to its affordable machinery and its capability to achieve high resolutions. Kotz et al. presented a wide range of 3D printable materials suitable for chemical synthesis applications 34. In general, polymers are the materials of choice in 3D printing due to their ease of processing and the fact that they can be structured using off‐the‐shelf, affordable instruments. However, for most polymers, properties such as liquid repellence, chemical and heat resistance remain critical, some of which are, unfortunately, often required for miniaturized chemical synthesis applications. Among the materials of choice for on‐chip synthesis are transparent silicate glasses, ceramics, and fluorinated polymers 34, 35, 36. Fluorinated polymers have been used in a variety of fields due to their excellent properties. They show high liquid (water/oil) repellence, high resistance towards chemicals and heat, chemical durability as well as outstanding solvent compatibility, to name a few 37. However, for microfluidic chemical synthesis on microchips, high optical transparency is required as well, mainly for the integration of online‐capable optical sensors and detectors.

In this work, we report the simple fabrication of a transparent microfluidic chip made of a perfluoropolyether dimethacrylate (PFPE‐MA) via 3D printing using SLA. The printed chips were characterized in terms of their optical, mechanical, and thermal properties, showing high transparency, high thermal and mechanical stability as well as outstanding resistance towards chemical solvent exposure. We demonstrate the suitability of this material and 3D printing as the manufacturing technology in chemistry‐on‐chip applications using two organic reactions: the discoloration of methylene blue and the synthesis of *N*‐benzylidenbenzylamin.

## 2 Experimental Work

### 2.1 Materials

Fluorolink MD700 (a perfluoropolyether (PFPE) dimethacrylate (MA)) was purchased from Acota (United Kingdom). *L*‐Ascorbic acid (>98 %), benzaldehyde (>99.5 %), benzylamine (>99.5 %), isopropylthioxanthone (ITX), phenylbis(2,4,6‐trimethylbenzoyl) phosphine oxide (PPO) and methylene blue (certified by biological stain commission) were purchased from Sigma‐Aldrich (Germany). Acetone (>99.5 %, synthesis grade), hydrochloric acid (37 % fuming, technical), methanol (HPLC gradient) and 2‐propanol were purchased from Carl Roth (Germany).

### 2.2 Preparation of the Resin

Prior to the preparation of the resin, a stock solution of the photoinitiator phenylbis(2,4,6‐trimethylbenzoyl) phosphine oxide (BAPO) and of the superabsorber ITX in acetone was prepared. 68 mg BAPO and 68 mg ITX were added to 1 mL of acetone. The prepared solution was mixed thoroughly. Afterwards, Fluorolink MD700 was mixed with the BAPO/ITX/acetone stock solution: 0.072 mL of the stock solution was added to 1 mL Fluorolink MD700. The resin was then mixed using an ultrasonic bath Sonorex Da300 (Bandelin electronic, Germany) for 5 min and finally degassed.

### 2.3 Fabrication of the Microfluidic Chips

The PFPE‐MA microfluidic chips were printed using a stereolithography printer Asiga Pico 2 (Asiga, Australia) with a wavelength of 385 nm and a light intensity of 88 W m^−2^. The first layer was chosen to be 200 µm and was exposed for 60 s to avoid delamination of the print from the platform. The thickness of the layers was set to 50 µm and each layer was exposed for 6 s. To avoid delamination of the layers during the process the z‐compensation was set to 200 µm. As Fluorolink MD700 has a high viscosity of 581 cP, the printing temperature was set to 45 °C to enable a better processability of the resin during the print. The printed PFPE‐MA microfluidic structures were developed in acetone for 5 min followed by sonication in an ultrasonic bath for 20 min at 45 °C to remove the uncured resin from the channels. Lastly, the chips were post‐cured for 2 min using UV‐radiation lamp (type Flash DR‐301C, Asiga, Australia).

### 2.4 Characterizations

*Thermogravimetric analysis (TGA)*: The thermal stability of the fabricated chips was assessed by thermogravimetric analysis (STA409C, Netzsch, Germany). The measurements were performed with a heating rate of 1 K min^−1^ at a temperature range from 25 °C to 700 °C.

*Ultraviolet‐ visible (UV‐VIS) measurements*: The optical transparency was measured using a UV‐Vis spectrometer (Evolution 201 from Thermo Scientific, Germany) on thin layers of the cured material (thickness 400 µm) using air as background reference.

*Compression test*: The mechanical stability of the printed microfluidic chips was assessed by compression tests. Pillars with 15 mm diameter and 10 mm thickness (for compression perpendicular to printing layers) and cubes with 15 mm edge length (for compression parallel to the printing layers) were 3D printed, washed with acetone, and dried overnight. Mechanical compression tests were conducted on an Inspect 5 instrument (Hegewald & Peschke, Germany). The printed pillars were measured in two different ways: by applying force perpendicular to the printing layers and by applying force parallel to the printing layers.

*Scanning electron microscopy (SEM)*: SEM images were taken with a Quanta 250 FEG (FEI Inc., USA). The acceleration potential was set to 5 kV. Samples were fixated on SEM‐sample holders with conductive tape. The sample surfaces were sputtered with a layer of gold‐palladium with a thickness of about 25 nm (Hummer 6.2 sputtering system from Anatech Ltd., USA).

*Optical Microscopy*: Optical microscopy was performed using a microscope of type VHX 6000 (Keyence Corporation, Japan) with a 20–100 magnification lens.

*Nuclear magnetic resonance (NMR)*: Proton nuclear magnetic resonance (^1^H‐NMR) spectra were recorded by an Avance NMR instrument (Bruker, USA) at 250 MHz. The sample was prepared in deuterated chloroform‐d with a concentration of 10 mg mL^−1^.

*Fourier transformed infrared (FTIR) measurements*: The characterization of the reaction products was conducted via an FTIR spectrometer (Frontier 100 MIR‐FTIR from Perkin Elmer, Germany) using an attenuated total reflection (ATR) measurement unit.

*Contact angle measurements*: Static water contact angles were measured with 10 µL sized water droplets with an OCA 15EC device (DataPhysics Instruments, Germany).

### 2.5 Microfluidic Chemical Assays

Two chemical reactions were tested to show the applicability of the printed microchips. Firstly, a discoloration reaction of methylene blue (MB) and *L‐*Ascorbic acid (Asc) in which MB reacts to its leuco form 23. For this, two solutions were prepared: an aqueous solution with 2.5 mmol L^−1^ of MB and 1 mol L^−1^ of hydrochloric acid and a solution with 0.28 mol L^−1^ of Asc in water. The second reaction was the synthesis of an imine (*N‐*benzylidenbenzylamin) from benzaldehyde and benzylamine in a methanolic solution. Two solutions of benzaldehyde and benzylamine in methanol with a concentration of 1 mol L^−1^ were prepared. The product was delivered through the outlet of the microfluidic chip and characterized by FTIR‐ATR and by ^1^H‐NMR measurements.

## 3 Results and Discussion

**Figure 1:**
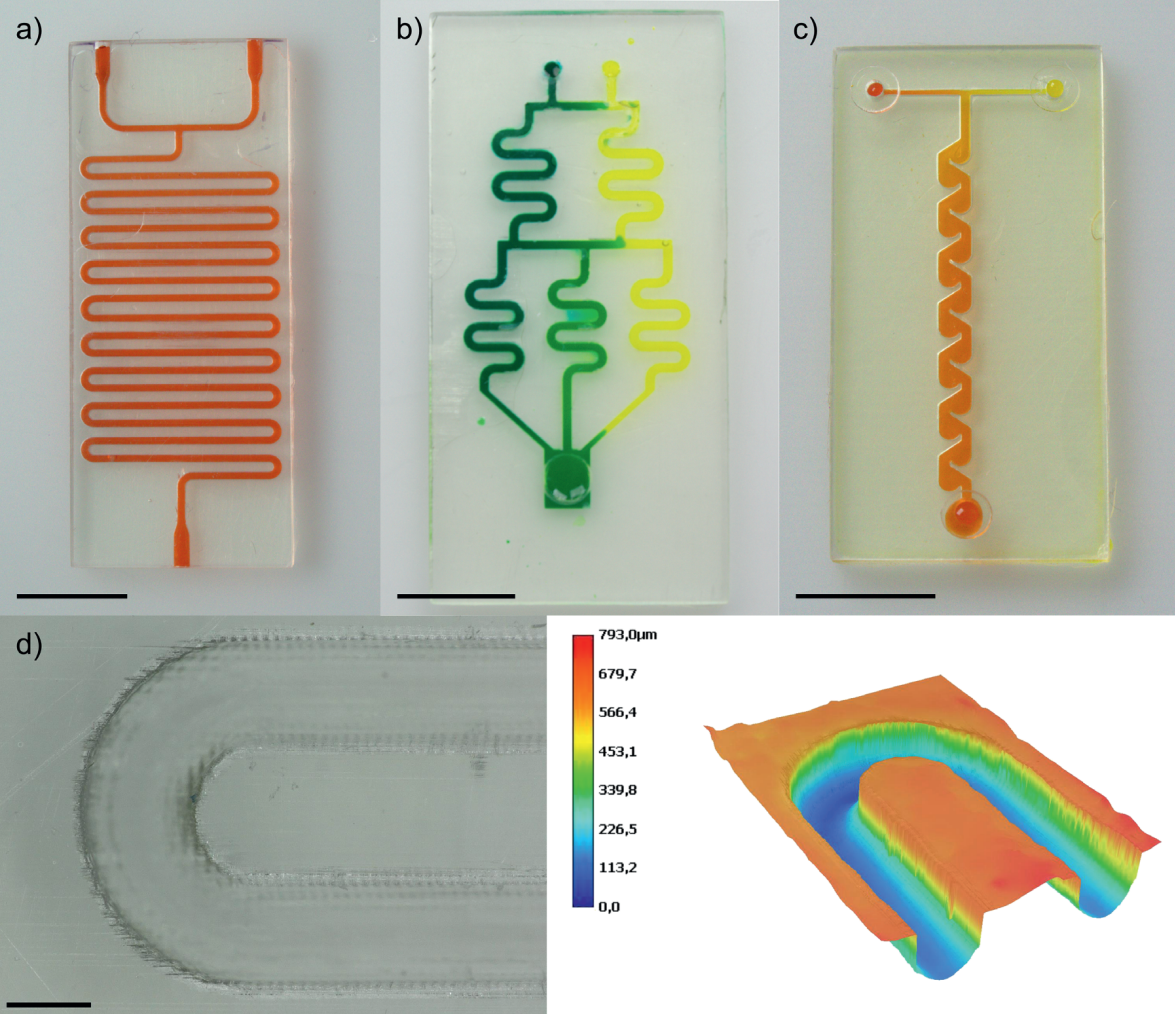
3D printed PFPE‐MA chips (channel widths ∼ 800 µm). a) Serpentine mixer filled with red dyed water. b) Gradient mixer filled with yellow and blue dyed water. c) Tesla mixer filled with red and yellow dyed water. Scale bar: 10 mm. d) Microscopical and topographical image of printed open channel with height information. The height of the half‐printed channel is confirmed to be around 400 µm. Scale bar: 1 mm.

Transparent PFPE‐MA microfluidic chips with a channel diameter of 800 µm were successfully printed using SLA with slicing thickness of 50 µm within less than 30 min. Fig. 1 shows three different PFPE‐MA chips that were manufactured: a serpentine microfluidic mixer, a gradient serpentine mixer, and a Tesla mixer showing the versatility of the printing method. The channels were filled with dyed water to show diffusive mixing. The gradient serpentine mixer in Fig. 1b shows efficient mixing of the blue dyed and yellow dyed water leading to a green color. The tesla mixer (Fig. 1c) shows the efficient mixing of yellow and red dyed water. To visualize the printed channels shapes and dimensions, optical microscopy measurements were performed on half‐printed channels (see Fig. 1d). The channels width was measured to be 792 ± 9 µm and thus close to the set width of 800 µm.

To further characterize the channels, SEM images were taken (Fig. S1 in the Supporting Information, SI). The cross‐section at horizontal as well as in the vertical cut planes were taken and show the typical stacked‐layer pattern, which results from the layer‐by‐layer printing process. We assessed the resolution of our printing method with a pattern of pillars and channels varying in size. The channels were printed perpendicular and parallel to the printing layers (Fig. S2). We reached 500 µm printing resolution for the pillars and the channels perpendicular to the printing layers as well as 600 µm printing resolution for the channels with the printing layers as the minimum feature size.

**Figure 2:**
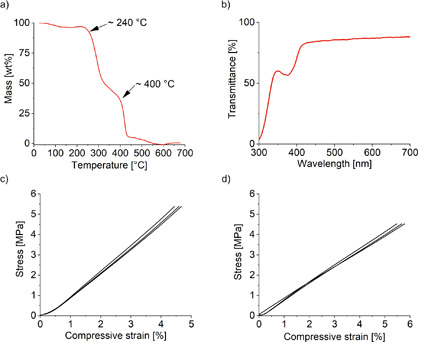
Characterization of PFPE‐MA chips: a) TGA measurement of 3D printed PFPE‐MA shows a first thermal decomposition at around 240 °C. b) UV‐VIS transmission spectra of a 400 µm thick 3D printed layer of PFPE‐MA showing a transparency of up to 90 % for visible light. c,d) Stress/strain curve for compressive testing perpendicular (c) and parallel (d) to the printing layers.

The contact angle measurements showed the hydrophobicity of the printed PFPE‐MA chips with a static water contact angle of 117 ± 1°. The thermal stability of the material is of interest for chemical reactions that, e.g., require heating or are strongly exothermic. TGA measurements of the printed chip show a primary weight loss at around 240 °C corresponding to the decomposition to hydrofluoric acid (a common product of the decomposition of fluorinated polymers) and formation of amorphous carbon (see Fig. 2a). A secondary weight loss was observed at around 400 °C corresponding to the thermal decomposition of the formed carbon. These results confirm a thermal stability of the printed chips up to around 200 °C. The found thermal decompositions are in good accordance with literature values for perfluoropolyethers 38. Fig. 2b shows the optical transmission of the printed chips. As can be seen, the chips show optical transmissions of 55–90 % over the range of 300–700 nm for a thickness of around 370 µm. Besides the optical properties, the mechanical properties of the chip are of high relevance as they define, e.g., how delicate the chip needs to be handled and if specialized mounting equipment is required. This is often the case, e.g., for glass or silicon microfluidic chips. We thus characterized the PFPE‐MA chips by performing compressive stress tests. The material is able to withstand a load of 950 N applied perpendicular to the printing layers without failure (Fig. 2c), which was the maximum applicable force of the measurement device. The material endured a final stress of 5.5 MPa with a compressive strain of 4.5 % resulting in a compressive Young's modulus of 1.27 ± 0.03 MPa. When compressive stress was applied parallel to the printing layers, no mechanical failure was observed up to 950 N load. The specimen endured a final stress of 4.5 MPa with a compressive strain of 5.6 % and a final Young's modulus of 0.79 ± 0.03 MPa (Fig. 2d). Such high mechanical endurance makes PFPE‐MA a good candidate for durable chemical synthesis applications.

**Figure 3:**
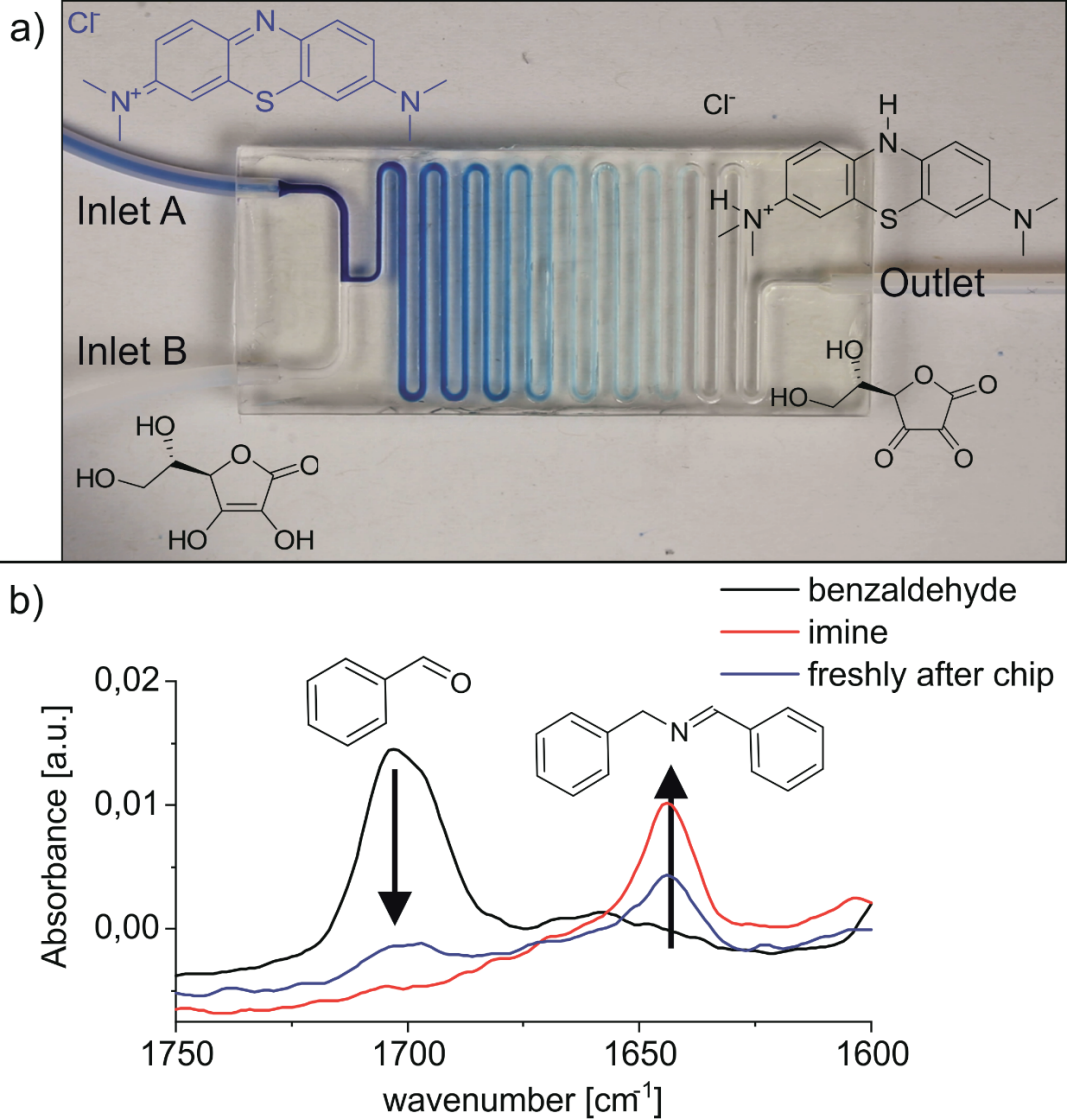
On‐chip chemical synthesis using the 3D printed PFPE‐MA chips: a) Reaction of MB with Asc resulting in a gradual discoloration of MB to its colorless leuco form. b) FTIR spectrum monitoring the synthesis of *N‐*benzylidenbenzylamin (imine) on‐chip showing the decreasing educt absorption peak and increasing imine product peak (1640 cm^−1^) as the reaction progresses. The black spectrum is the pure educt (benzaldehyde), first collected solution right after the mixing (blue) and the collected product 5 min after the reaction was performed (red).

We have previously demonstrated the outstanding chemical stability and solvent compatibility of printed PFPE‐MA, which allows reactions in almost all common organic solvents 39. To this effect, we implemented two on‐chip reactions building on the excellent mechanical stability, high optical transparency and high solvent compatibility of the printed chips. The first reaction involves the reaction of methylene blue to its colorless leuco form. The second reaction was the on‐chip synthesis of *N‐*benzylidenbenzylamin, as an imine. Imines are well‐known intermediate products in organic synthesis for their reactivity and versatility in many organic reactions 40 as synthesis educts in pharmaceuticals 41, 42 and anti‐inflammatory drugs 43. These reactions were selected to demonstrate the broad application range of this 3D printed chip material. Therefore, one reaction in aqueous media and one reaction in organic solvent, namely methanol, were chosen to show the chemical resistance of the material. Both reactions can be easily monitored as the reaction of methylene blue with ascorbic acid is observed by visual discoloration of the solution through the transparent chip material, and the synthesis of *N*‐benzylidenbenzylamin at ambient conditions was presented by FTIR measurements. The reactions were conducted on a microfluidic reactor with a T‐junction and a serpentine mixing structure, which was 3D printed. The chip with a circular channel diameter of 800 µm with a lateral dimension of 20 × 48 mm^2^ is shown in Fig. 3a.

For both reactions the educts were pumped via syringe pumps (Legato® 110, KDScientific, USA) with 50 µL min^−1^ flowrate through PTFE‐tubing with 0.5 mm inner diameter (Bola, Germany) into the microfluidic chip. The outlet was connected via a PTFE‐tube to a collection vial. In the first reaction, the blue colored MB solution reacted with Asc showing a gradual discoloration throughout the channel to the colorless leuco form (see Fig. 3a). In the second reaction, the same chip design and setup was used for the synthesis of *N*‐benzylidenbenzylamin (imine) from benzaldehyde and benzylamine in methanol 23. The synthesis was monitored by FTIR to confirm the formation of the imine as all educts are colorless. The FTIR measurements were conducted at two points in time: The first one was made with collected solution right after the reaction started (blue spectrum) and the second one after around 5 min from running the reaction (red spectrum). The presented FTIR shown in Fig. 3b confirms the progress of these reaction over time. As the benzaldehyde reacts with the benzylamine forming the imine, the absorption peak at 1700 cm^−1^ of the benzaldehyde decreases and the peak at 1640 cm^−1^ of the formed imine increases. To further characterize the reaction product ^1^H‐NMR measurement was conducted. The NMR spectra showed all expected peaks for the imine (see SI).

## 4 Conclusion

We showed the simple fabrication of microfluidic chips with a circular channel diameter of 800 µm via SLA‐based 3D printing using PFPE‐MA, a highly fluorinated perfluoropolyether dimethacrylate. Different designs, such as a serpentine mixer, a Tesla mixer, and a gradient mixer were printed successfully. The printed chips show high transmissions up to 90 % for visible light and a high thermal stability up to 200 °C. These chips show high mechanical resilience towards compressive strain and no mechanical failure was observed up to 950 N load. All of these characteristics make PFPE‐MA a very promising material for on‐chip chemical synthesis applications. We tested two chemical reactions on the chips, the discoloration of methylene blue and the synthesis of *N*‐benzylidenbenzylamin, both confirming the suitability of 3D printed PFPE‐MA for on‐chip synthesis platforms. We believe that the combination of the developed PFPE‐MA with its ease of fabrication will pave the way for a wide range of on‐chip chemical synthesis applications making use of the many advantages inherent to microfluidics.

## 5 Supporting Information

Supporting Information for this article can be found under DOI: https://doi.org/10.1002/cite.202200013.

## Abbreviations

^1^H‐NMR: proton nuclear magnetic resonance
3D: three dimensional
Asc: *L*‐ascorbic acid
BAPO: phenylbis(2,4,6‐trimethylbenzoyl) phosphine oxide
FTIR: Fourier transformed infrared
FTIR‐ATR: Fourier transformed infrared ‐ attenuated total reflection
HPLC: high performance liquid chromatography
ITX: isopropylthioxanthone
LOC: lab on a chip
MB: methylene blue
PFPE: perfluoropolyether
PFPE‐MA: perfluoropolyether‐dimethacrylate
SEM: scanning electron microscopy
SLA: stereolithography apparatus
TGA: thermogravimetric analysis
UV: ultraviolet
UV‐VIS: ultraviolet‐visible

## Supporting information

Supplementary Information

## References

[1] A.‐C. Bédard , A. Adamo , K. C. Aroh , M. G. Russell , A. A. Bedermann , J. Torosian , B. Yue , K. F. Jensen , T. F. Jamison , Reconfigurable system for automated optimization of diverse chemical reactions, Science 2018, 361, 1220–1225. DOI: 10.1126/science.aat0650 30237351

[2] P. Y. Keng , S. Chen , H. Ding , S. Sadeghi , G. J. Shah , A. Dooraghi , M. E. Phelps , N. Satyamurthy , A. F. Chatziioannou , C.‐J. C. Kim , R. M. van Dam , Micro‐chemical synthesis of molecular probes on an electronic microfluidic device, Proc. Natl. Acad. Sci. U. S. A. 2012, 109, 690–695. DOI: 10.1073/pnas.1117566109 22210110 PMC3271918

[3] S. S. Zalesskiy , E. Danieli , B. Blümich , V. P. Ananikov , Miniaturization of NMR Systems: Desktop Spectrometers, Microcoil Spectroscopy, and “NMR on a Chip” for Chemistry, Biochemistry, and Industry, Chem. Rev. 2014, 114, 5641–5694. DOI: 10.1021/cr400063g 24779750

[4] J. A. Schwartz , J. V. Vykoukal , P. R. C. Gascoyne , Droplet‐based chemistry on a programmable micro‐chip, Lab Chip 2004, 4, 11. DOI: 10.1039/b310285h 15007434 PMC2726250

[5] I. R. Baxendale , The integration of flow reactors into synthetic organic chemistry: Integration of flow reactors into synthetic organic chemistry, J. Chem. Technol. Biotechnol. 2013, 88, 519–552. DOI: 10.1002/jctb.4012

[6] F. Grisoni et al., Combining generative artificial intelligence and on‐chip synthesis for de novo drug design, Sci. Adv. 2021, 7 (24). DOI: 10.1126/sciadv.abg3338 PMC8195470 34117066

[7] T. Schwalbe , V. Autze , G. Wille , Chemical Synthesis in Microreactors, Chimia 2002, 56 (11), 636. DOI: 10.2533/000942902777679984

[8] F. Mi , C. Hu , Y. Wang , L. Wang , F. Peng , P. Geng , M. Guan , Recent advancements in microfluidic chip biosensor detection of foodborne pathogenic bacteria: a review, Anal. Bioanal. Chem. 2022, 414, 2883. DOI: 10.1007/s00216-021-03872-w 35064302 PMC8782221

[9] R. S. Ramsey , J. M. Ramsey , Generating Electrospray from Microchip Devices Using Electroosmotic Pumping, Anal. Chem. 1997, 69 (6), 1174–1178. DOI: 10.1021/ac9610671 21639399

[10] M. Fani , P. Pourafshary , P. Mostaghimi , N. Mosavat , Application of microfluidics in chemical enhanced oil recovery: A review, Fuel 2022, 315, 123225. DOI: 10.1016/j.fuel.2022.123225

[11] E. Verpoorte , Microfluidic chips for clinical and forensic analysis, Electrophoresis 2002, 23, 677–712. DOI: 10.1002/1522-2683(200203)23:5<677::AID-ELPS677>3.0.CO;2-8 11891702

[12] G. M. Whitesides , The origins and the future of microfluidics, Nature 2006, 442, 368–373. DOI: 10.1038/nature05058 16871203

[13] G. Weisgrab , A. Ovsianikov , P. F. Costa , Functional 3D Printing for Microfluidic Chips, Adv. Mater. Technol. 2019, 4, 1900275. DOI: 10.1002/admt.201900275

[14] S. Waheed , J. M. Cabot , N. P. Macdonald , T. Lewis , R. M. Guijt , B. Paull , M. C. Breadmore , 3D printed microfluidic devices: enablers and barriers, Lab Chip 2016, 16, 1993–2013. DOI: 10.1039/C6LC00284F 27146365

[15] N. Bhattacharjee , A. Urrios , S. Kang , A. Folch , The upcoming 3D‐printing revolution in microfluidics, Lab Chip 2016, 16, 1720–1742. DOI: 10.1039/C6LC00163G 27101171 PMC4862901

[16] N. Xiang , H. Yi , K. Chen , S. Wang , Z. Ni , Investigation of the maskless lithography technique for the rapid and cost‐effective prototyping of microfluidic devices in laboratories, J. Micromech. Microeng. 2013, 23, 025016. DOI: 10.1088/0960-1317/23/2/025016

[17] M. Focke , D. Kosse , C. Müller , H. Reinecke , R. Zengerle , F. von Stetten , Lab‐on‐a‐Foil: microfluidics on thin and flexible films, Lab Chip 2010, 10, 1365–1386. DOI: 10.1039/C001195A 20369211

[18] U. M. Attia , S. Marson , J. R. Alcock , Micro‐injection moulding of polymer microfluidic devices, Microfluid Nanofluid 2009, 7, 1. DOI: 10.1007/s10404-009-0421-x

[19] A. Manz , J. C. Fettinger , E. Verpoorte , H. Lüdi , H. M. Widmer , D. J. Harrison , Micromachining of monocrystalline silicon and glass for chemical analysis systems A look into next century's technology or just a fashionable craze?, TrAC, Trends Anal. Chem. 1991, 10, 144–149. DOI: 10.1016/0165-9936(91)85116-9

[20] D. J. Harrison , A. Manz , Z. Fan , H. Luedi , H. M. Widmer , Capillary electrophoresis and sample injection systems integrated on a planar glass chip, Anal. Chem. 1992, 64, 1926–1932. DOI: 10.1021/ac00041a030

[21] D. C. Duffy , J. C. McDonald , O. J. A. Schueller , G. M. Whitesides , Rapid Prototyping of Microfluidic Systems in Poly(dimethylsiloxane), Anal. Chem. 1998, 70, 4974–4984. DOI: 10.1021/ac980656z 21644679

[22] A. R. Wheeler , W. R. Throndset , R. J. Whelan , A. M. Leach , R. N. Zare , Y. H. Liao , K. Farrell , I. D. Manger , A. Daridon , Microfluidic Device for Single‐Cell Analysis, Anal. Chem. 2003, 75, 3581–3586. DOI: 10.1021/ac0340758 14570213

[23] P. J. Kitson , M. H. Rosnes , V. Sans , V. Dragone , L. Cronin , Configurable 3D‐Printed millifluidic and microfluidic ‘lab on a chip’ reactionware devices, Lab Chip. 12 (2012) 3267–3271. DOI: 10.1039/C2LC40761B 22875258

[24] P. J. Kitson , R. J. Marshall , D. Long , R. S. Forgan , L. Cronin , 3D Printed High‐Throughput Hydrothermal Reactionware for Discovery, Optimization, and Scale‐Up, Angew. Chem., Int. Ed. 2014, 53, 12723–12728. DOI: 10.1002/anie.201402654 25079230

[25] G. I. J. Salentijn , H. P. Permentier , E. Verpoorte , 3D‐Printed Paper Spray Ionization Cartridge with Fast Wetting and Continuous Solvent Supply Features, Anal. Chem. 2014, 86, 11657–11665. DOI: 10.1021/ac502785j 25409532

[26] G. W. Bishop , J. E. Satterwhite , S. Bhakta , K. Kadimisetty , K. M. Gillette , E. Chen , J. F. Rusling , 3D‐Printed Fluidic Devices for Nanoparticle Preparation and Flow‐Injection Amperometry Using Integrated Prussian Blue Nanoparticle‐Modified Electrodes, Anal. Chem. 2015, 87, 5437–5443. DOI: 10.1021/acs.analchem.5b00903 25901660 PMC4439300

[27] W. Su , B. S. Cook , Y. Fang , M. M. Tentzeris , Fully inkjet‐printed microfluidics: a solution to low‐cost rapid three‐dimensional microfluidics fabrication with numerous electrical and sensing applications, Sci. Rep. 2016, 6, 35111. DOI: 10.1038/srep35111 27713545 PMC5054388

[28] R. Brossard , T. Brouchet , F. Malloggi , Replication of a Printed Volatile Mold: a novel microfabrication method for advanced microfluidic systems, Sci. Rep. 2019, 9, 17473. DOI: 10.1038/s41598-019-53729-7 31767890 PMC6877523

[29] V. Bertana , C. Potrich , G. Scordo , L. Scaltrito , S. Ferrero , A. Lamberti , F. Perrucci , C. F. Pirri , C. Pederzolli , M. Cocuzza , S. L. Marasso , 3D‐printed microfluidics on thin poly(methyl methacrylate) substrates for genetic applications, J. Vac. Sci. Technol., B 2017, 36, 01A106. DOI: 10.1116/1.5003203

[30] B. M. de C. Costa , S. Griveau , F. Bedioui , F. d'Orlye , J. A. F. da Silva , A. Varenne , Stereolithography based 3D‐printed microfluidic device with integrated electrochemical detection, Electrochim. Acta 2022, 407, 139888. DOI: 10.1016/j.electacta.2022.139888

[31] A. V. Nielsen , M. J. Beauchamp , G. P. Nordin , A. T. Woolley , 3D Printed Microfluidics, Annu. Rev. Anal. Chem. 2020, 13 (1), 45–65. DOI: 10.1146/annurev-anchem-091619-102649 PMC7282950 31821017

[32] A. Waldbaur , H. Rapp , K. Länge , B. E. Rapp , Let there be chip—towards rapid prototyping of microfluidic devices: one‐step manufacturing processes, Anal. Methods 2011, 3, 2681–2716. DOI: 10.1039/C1AY05253E

[33] Y. He , Y. Wu , J. Fu , Q. Gao , J. Qiu , Developments of 3D Printing Microfluidics and Applications in Chemistry and Biology: a Review, Electroanalysis 2016, 28, 1658–1678. DOI: 10.1002/elan.201600043

[34] F. Kotz , P. Risch , D. Helmer , B. E. Rapp , High‐Performance Materials for 3D Printing in Chemical Synthesis Applications, Adv. Mater. 2019, 31, 1805982. DOI: 10.1002/adma.201805982 30773705

[35] A. de Mello , Focus: Plastic fantastic?, Lab Chip 2002, 2, 31N. DOI: 10.1039/b203828p 15100832

[36] F. Kotz , K. Arnold , S. Wagner , W. Bauer , N. Keller , T. M. Nargang , D. Helmer , B. E. Rapp , Liquid PMMA: A High Resolution Polymethylmethacrylate Negative Photoresist as Enabling Material for Direct Printing of Microfluidic Chips, Adv. Eng. Mater. 2018, 20, 1700699. DOI: 10.1002/adem.201700699

[37] H. Teng , Overview of the Development of the Fluoropolymer Industry, Appl. Sci. 2012, 2, 496–512. DOI: 10.3390/app2020496

[38] C. M. Baxter , J. McCollum , S. T. Iacono , Preparation and Thermal Analysis of Blended Nanoaluminum/Fluorinated Polyether‐Segmented Urethane Composites, J. Compos. Sci. 2019, 3, 25. DOI: 10.3390/jcs3010025

[39] F. Kotz , P. Risch , D. Helmer , B. E. Rapp , Highly Fluorinated Methacrylates for Optical 3D Printing of Microfluidic Devices, Micromachines 2018, 9, 115. DOI: 10.3390/mi9030115 PMC6187856 30424049

[40] J. S. M. Samec , A. H. Éll , J.‐E. Bäckvall , Efficient Ruthenium‐Catalyzed Aerobic Oxidation of Amines by Using a Biomimetic Coupled Catalytic System, Chem. – Eur. J. 2005, 11, 2327–2334. DOI: 10.1002/chem.200401082 15706621

[41] Y. Yamamoto , Y. Watanabe , S. Ohnishi , 1,3‐Oxazines and Related Compounds. XIII. Reaction of Acyl Meldrum's Acids with Schiff Bases Giving 2,3‐Disubstituted 5‐Acy1‐3,4,5,6‐tetrahydro‐2H‐1, 3‐oxazine‐4,6‐diones and 2,3,6‐Trisubstituted 2,3‐Dihydro‐1,3‐oxazin‐4‐ones, Chem. Pharm. Bull. 1987, 35, 1860–1870. DOI: 10.1248/cpb.35.1860

[42] A. Studer , P. Jeger , P. Wipf , D. P. Curran , Fluorous Synthesis: Fluorous Protocols for the Ugi and Biginelli Multicomponent Condensations, J. Org. Chem. 1997, 62, 2917–2924. DOI: 10.1021/jo970095w 11671655

[43] D. J. Hadjipavlou‐Litina , A. A. Geronikaki , Thiazolyl and benzothiazolyl Schiff bases as novel possible lipoxygenase inhibitors and anti inflammatory agents. Synthesis and biological evaluation, Drug Des. Discovery 1998, 15, 199–206. 9689502

