# Supplementary material for: On‐Chip Chemical Synthesis Using One‐Step 3D Printed Polyperfluoropolyether
Source: Chem Ing Tech. 2022 Apr 25;94(7):975–82. doi: 10.1002/cite.202200013 (PMC9322562; doi:10.1002/cite.202200013)
Supplement: Supplementary file 1 — Supplementary Information [file CITE-94-975-s001.pdf]

Supporting Information

On-Chip Chemical Synthesis Using One-Step 3D Printed Polyperfluoropolyether

Andreas Goralczyk, Fadoua Mayoussi, Mario Sanjaya, Santiago Franco Corredor, Sagar Bhagwat, Qingchuan Song, Sarah Schwenteck, Andreas Warmbold, Pegah Pezeshkpour, Bastian E. Rapp

DOI: 10.1002/cite.202200013

Correspondence: Pegah Pezeshkpour (Pegah.Pezeshkpour@neptunlab.org), Georges-Koehler Allee 103, 79110 Freiburg, Germany.

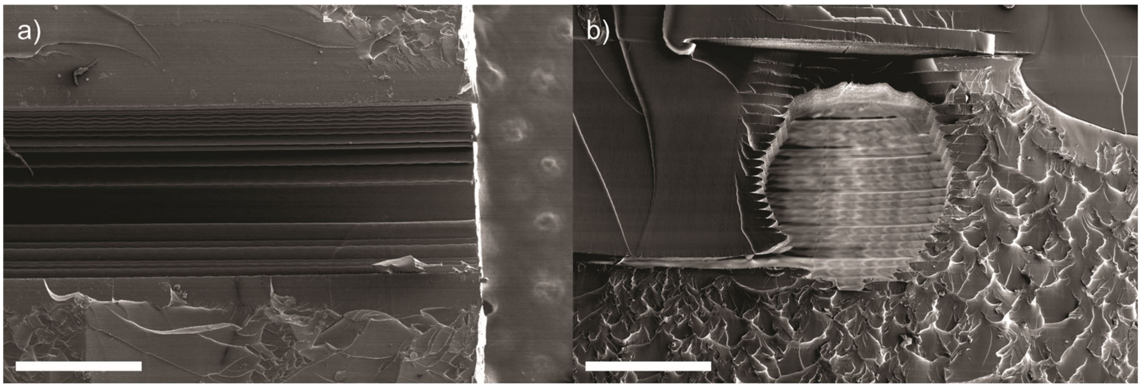

Figure S1. Scanning electron microscopy images of an open 3D printed microfluidic channel made from PFPE-MA: a) Cross-section view perpendicular to the build direction and b) cross-section view parallel to the build direction of channel with 800 µm diameter. Scale bar: 500 µm.

For the investigation of the printing resolution a pattern pillar and channel arrays were printed. Figure S2 shows the microscopical images of the printed pillars and channels perpendicular to and within the printing plane.

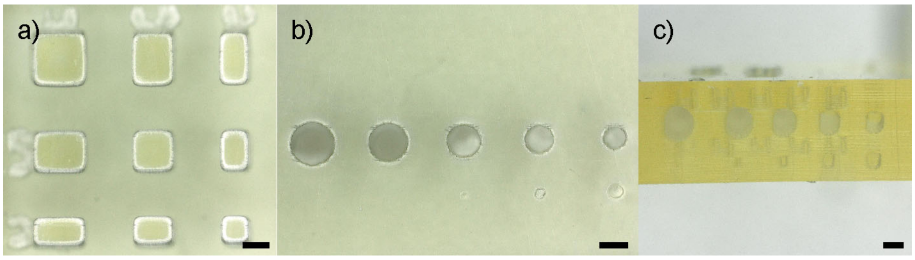

Figure S 2. Microscopical images of 3D-printed patterns for the assessment of the printing resolution. a) Pillars could be printed up to a size of 500 µm feature size. b) Channels printed perpendicular to the printing layers with 500 µm diameter, and c) channels printed parallel to the printing layers with 600 µm diameter. Scale bar: 500 µm.

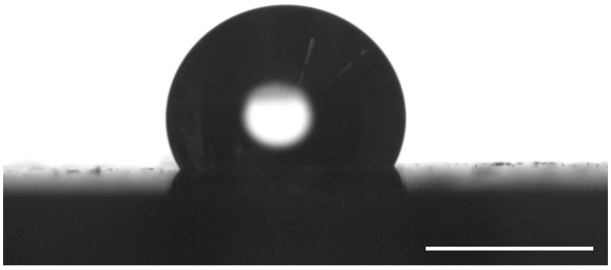

Figure S 3. Water droplet (10 µL) on top of a PFPE-MA chip with a static contact angle of 117 ± 1°. Scale bar: 1 mm.

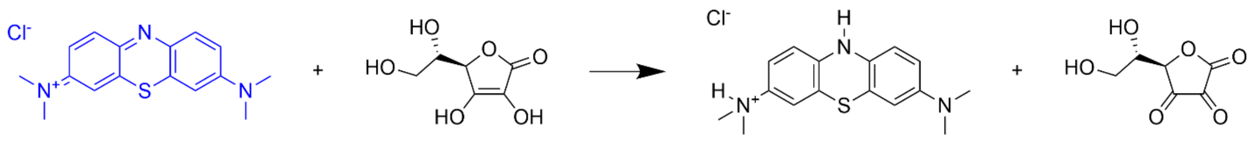

Scheme S1. Reaction scheme of methylene blue and ascorbic acid to leuco methylene blue and *L*-dehydro ascorbic acid.

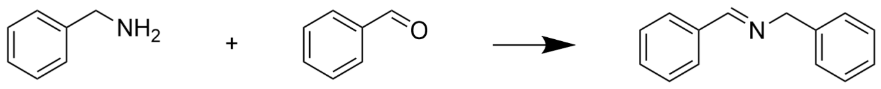

Scheme S2. Reaction scheme of benzylamine and benzaldehyde to *N*-benzylidenbezylamin.

The synthesis of *N*-benzylidenbezylamin (imine) was monitored by FTIR measurements. **IR:** the peak at 1700 cm^{-1} is from the C=O moiety belonging to benzaldehyde and the peak at 1640 cm^{-1} is from the C=N-C functional group of the imine. The product solution was isolated and the solvent evaporated prior to NMR analysis. The ^{1}H-NMR spectrum is presented in **Figure S4**. ^{1}H-NMR (250 MHz, $CDCl_3$) (δ, ppm): 8.316 (s, 1H), 7.703 (m, 2H), 7.339 (m, 3H), 7.257 (m, 4H), 7.176 (m, 1H), 4.747 (s, 2H)

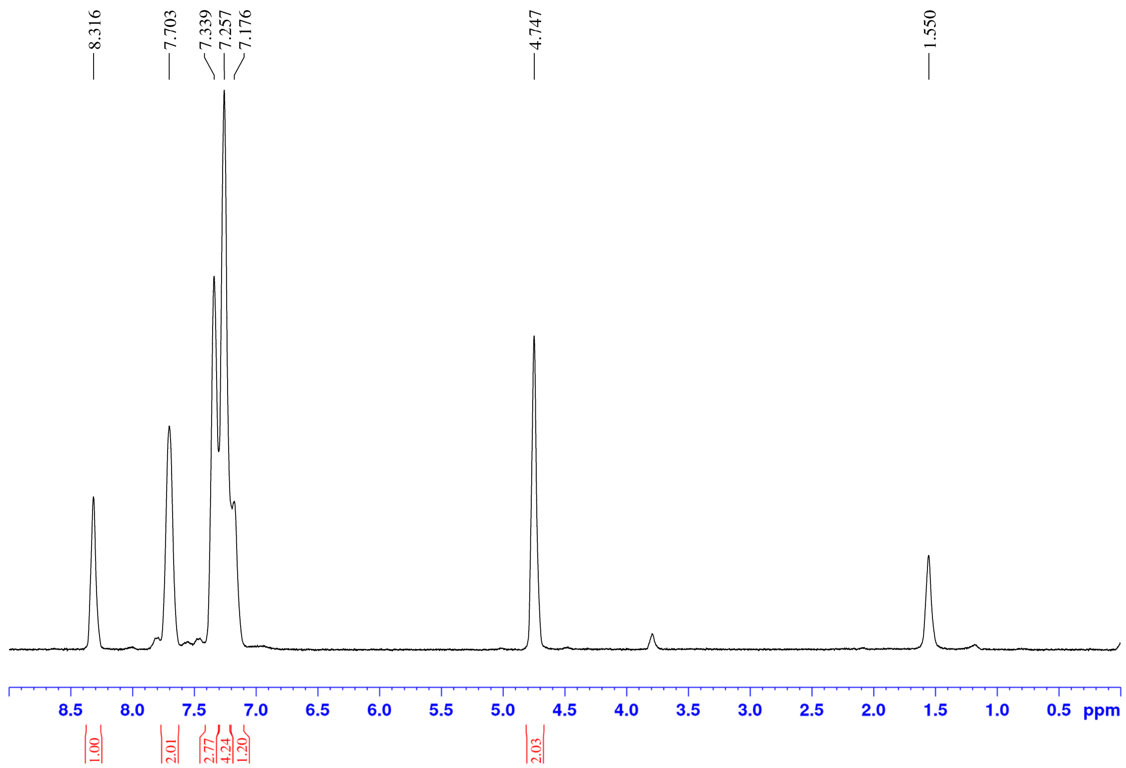

Figure S4. 1H-NMR spectrum of synthesized *N*-benylidenbenzylamin measured in chloroform-d.
